# Quantitative evaluation of frequency domain measurements in high density diffuse optical tomography

**DOI:** 10.1117/1.JBO.26.5.056001

**Published:** 2021-05-04

**Authors:** Guy A. Perkins, Adam T. Eggebrecht, Hamid Dehghani

**Affiliations:** a University of Birmingham, Sci-Phy-4-Health Centre for Doctoral Training, College of Engineering and Physical Sciences, Birmingham, United Kingdom; b University of Birmingham, College of Engineering and Physical Sciences, School of Computer Science, Birmingham, United Kingdom; c Washington University School of Medicine, Mallinckrodt Institute of Radiology, St. Louis, Missouri, United States

**Keywords:** frequency domain, modulation frequency, near-infrared spectroscopy, diffuse optical tomography, brain imaging, resolution

## Abstract

**Significance:** High density diffuse optical tomography (HD-DOT) as applied in functional near-infrared spectroscopy (fNIRS) is largely limited to continuous wave (CW) data. Using a single modulation frequency, frequency domain (FD) HD-DOT has recently demonstrated better localization of focal activation as compared to CW data. We show that combining CW and FD measurements and multiple modulation frequencies increases imaging performance in fNIRS.

**Aim:** We evaluate the benefits of multiple modulation frequencies, combining different frequencies as well as CW data in fNIRS HD-DOT.

**Approach:** A layered model was used, with activation occurring within a cortex layer. CW and FD measurements were simulated at 78, 141, and 203 MHz with and without noise. The localization error, full width half maximum, and effective resolution were evaluated.

**Results:** Across the average of the three metrics, at 141 MHz, FD performed 8.4% better than CW, and the combination of CW and FD was 21.7% better than CW. FD measurements at 203 MHz performed 5% better than 78 MHz. Moreover, the three combined modulation frequencies of FD and CW performed up to 3.92% better than 141 MHz alone.

**Conclusions:** We show that combining CW and FD measurements offers better performance than FD alone, with higher modulation frequencies increasing accuracy. Combining CW and FD measurements at multiple modulation frequencies yields the best overall performance.

## 1 Introduction

Functional near-infrared spectroscopy (fNIRS) is a medical imaging modality that can be used to monitor and diagnose many different pathologies within the brain. Such pathologies include but are not limited to Alzheimer’s,^1^ Parkinson’s,^2^ epilepsy,^3^ and traumatic brain injury.^4^^,^^5^ Psychiatric conditions such as schizophrenia^6^ and anxiety^7^ have also been studied, demonstrating the benefit of this technique over conventional technologies such as fMRI, PET, and EEG. The basic principle behind fNIRS is that different constituents in biological tissue absorb and scatter near-infrared (NIR) wavelengths of light (typically 650 to 950 nm) to a varying degree based on their extinction coefficient, cellular size, and density. By transmitting NIR light into soft tissue and measuring the scattered output, different functional properties of soft tissue can therefore be obtained through the use of model-based analysis and image reconstruction. Traditionally, fNIRS uses sparse arrays of sources and detectors to recover spectroscopic information, which are then often used with simplistic models based on the Beer–Lambert Law to derive the bulk concentration of a chromophore, typically oxy and deoxy hemoglobin.^8^ Using a high density grid of sources and detectors, tomographic image recovery of spatial distributions of optical properties has been shown to provide maps of localized concentration of these chromophores, which for functional cerebral imaging is known to be highly useful.^9^^,^^10^

There are three different methods and technologies for fNIRS data collection, namely continuous wave (CW), frequency domain (FD), and time domain (TD). CW is the most commonly adopted approach, which is achieved by detecting a change in the intensity of light, due to photons being absorbed or scattered in a medium. It is well established that CW can only provide attenuation-related changes in tissue as no information about the pathlength can be measured and is often assumed, depending on the tissue being imaged. FD-based systems provide a second parameter of light transport, namely the phase of photon propagation that reflects the average time taken for light to travel from a source to a given detector. The intensity of light transmitted in FD is sinusoidally modulated at a given frequency f (the modulation frequency, typically 100s MHz), with the measured light having three properties: AC is the amplitude of the intensity of oscillations, DC is the average intensity of light oscillations, and ϕ is the phase. The phase of the detected light therefore provides information regarding how much it has been delayed (on average) as a result of electromagnetic interactions with a medium, i.e., scattering. The phase is directly related to the time delay of light, τ, such that ϕ is in the order of ωτ, where ω=2πf and ω is the angular modulation frequency. For a good signal-to-noise ratio, it is required that ωτ≈1 (radians).^11^^,^^12^ If ωτ was ≪1, there would not be a measurable phase change. If ωτ was ≫1, then the AC amplitude decreases and could be below the level of noise. In the context of imaging biological tissue, time delays are on the order of nanoseconds (for source–detector separations of centimeters), which yields modulation frequencies in the order of a 100 MHz. For an FD system, the measured signal is described in Eq. (1),^13^ which for ω=0 yields the CW case, which is just the DC amplitude:

$$\text{Signal}=\mathrm{DC}+\mathrm{AC}e^{i\phi }e^{-i\omega t}.$$ (1)

TD technologies, often considered the gold standard, measure the time of flight of photons in a medium due to pulses of light (typically at a few tens of picoseconds), to build a histogram of the detected temporal point spread function, from which both absorption and scattering properties of tissue can be derived. For a review of TD technologies, a detailed overview can be found elsewhere.^14^

To allow functional imaging using fNIRS, the data need to be reliable and provide reasonable spatial and/or time resolution. The time resolution of fNIRS is determined by the sampling rate of the instrument: for example, the ISS Imagent device (ISS Inc., Champaign, Illinois) has a sampling rate of ∼40  Hz.^10^ The spatial resolution is itself a function of source–detector arrangement^15^ and the type of data^10^ (CW, FD, or TD). This work will evaluate the effect of modulation frequency and the combination of data types on the spatial resolution and accuracy via image quality assessment.

fNIRS studies are typically performed using a sparse set of sources and detectors, using CW measurements.^16^^,^^17^ Although the development of fNIRS can be dated to as early as the 1990s, it was not until the early 2000s where high density diffuse optical tomography (HD-DOT) was used.^18^ For example, in 2007, retinotopic mapping of adult human visual cortex was performed using a high density grid of 24 sources and 28 detectors on the visual cortex, which showed improved accuracy over previous studies.^19^ The performance increase of the CW HD array was quantified in White and Culver’s study in 2010,^15^ where they simulated cortical activation in a two-layered head model. They found that the HD grid was not only more accurate than a triangular or sparse array [over 50% better for effective resolution (ERES), 13 mm compared to 31 mm (triangular) and 29 mm (sparse)] but also provided more uniform localization across the imaging array.

Although FD data have been utilized previously for the recovery of scattering related changes, such as those of event-related optical signal, the major advancement to date has been the utilization of FD measurements for recovery of the absorption related (vascular) signals. The use of FD-NIRS has been previously demonstrated and review of the measurement technique has been extensively reported;^20^ however, there has been no quantification of the performance benefits of FD-NIRS for recovery of vascular-related focal activation signals until 2019.^10^ Doulgerakis et al. showed that FD measurements, through both amplitude and phase of the measured signal, offer an improved ERESs than CW measurements for second (29 mm) to fourth (47 mm) nearest neighbor source–detector separations, both with and without noise. The performance of FD has been shown to be better than CW as depth increases in human head model: recovery from cortical activity shows that FD can recover cortical activity whereas CW shows the same recovery at superficial tissue.

A more recent advancement is through the concept of using dual slopes for FD NIRS measurements.^21^ A normal source–detector (S-D) measurement simply provides the signal at one detector from a given source. A single slope method considers a single source and two detectors (such as those often utilized by the Hamamtsu NIRO systems), whereas the dual slope is defined as the average of two single slopes, such that the distance from a given source and detector is the same as those from another source and detector pair. One of the major advantages of the dual slope method is that it mathematically cancels the contributions of measurement error due to source–detector placement, detector drift, or any other unwanted temporal fluctuations during an experiment. The dual slope method with two sources and two detectors has shown to offer maximal sensitivity at a depth of 5 and 11 mm for amplitude and phase measurements, respectively, under typical conditions of fNIRS blood-perfused tissue, as compared to <2  mm and <5  mm for single distance S-D arrangements.

As outlined above, FD measurements using phase data have been shown to offer better performance in spatial resolution and depth sensitivity and are less effected by superficial tissue, highlighting the fact that it is advantageous to use FD measurements in fNIRS and HD-DOT. As many studies use CW, FD or TD measurements alone, or the combination of CW and FD, to date, no work has quantitatively compared the performance of CW versus FD versus CW and FD data. This work will present an objective and comprehensive evaluation of the performance between these data types as applied in HD-DOT.

Additionally as the FD measurements can be made at different modulation frequencies, with these typically being on the order of 100 MHz, little work has been done on the evaluation of the difference in performance due to different modulation frequencies. It is known that inclusion of FD data can increase sampling depth^10^ and that the magnitude of phase measurements has a modulation frequency dependency as seen in Eq. (1), therefore making the modulation frequency an interesting parameter to investigate.

There are three main objectives that will be addressed in this work for the application of FD-HD-DOT. The first is to quantify the performance of CW versus FD versus CW&FD. The second is to evaluate the effects of using different modulation frequencies and the third is to evaluate the effect of combining measurements at multiple modulation frequencies. Methods of the computational work are explained in Sec. 2 with the results presented in Sec. 3.

## 2 Methods

**Fig. 1:**
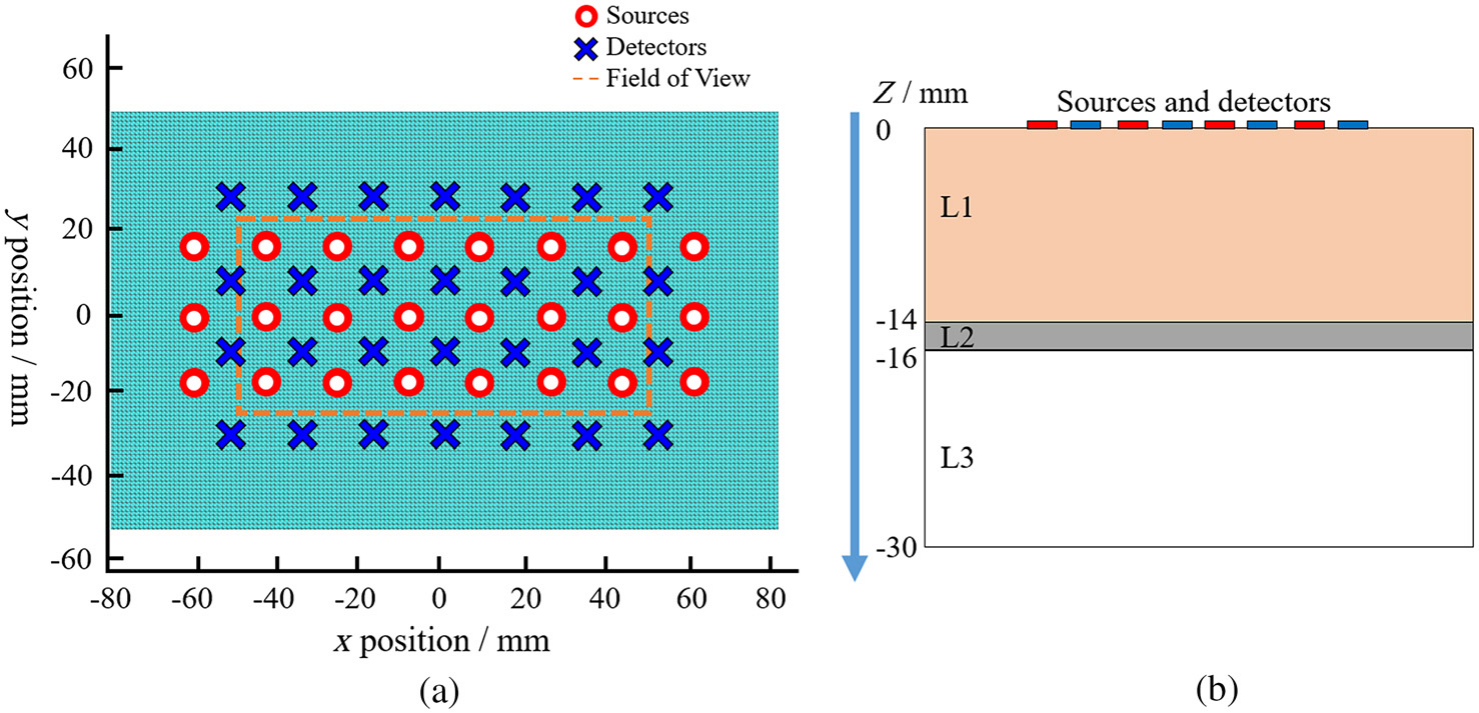
(a) A top down view of the HD grid FEM. There are 24 sources (blue crosses) and 28 detectors (red circles). (b) A schematic view in the Z plane of the FEM depicting three different layers. Layer 1 represents the skin, scalp, and CSV. Layer 2 represents gray matter and layer 3 represents white matter. The absorption and reduced scattering coefficients of the three layers are shown in Table 1.

To replicate the layers sampled in the human head, a simple three layer finite element model (FEM) was created using the near-infrared fluorescence and spectral tomography) software package,^22^ Figs. 1(a) and 1(b). This simplified model was used to allow a direct comparison to previous work outlining the benefits of HD-DOT, as well as providing a robust geometry for analysis of the results.^15^ The mesh has dimensions of 160, 100, and 30 mm in x, y, and z, respectively. The nodal resolution of the FEM is 1 mm, giving a total of 504,091 nodes (blue dots), corresponding to 2,880,000 linear tetrahedral elements. The three layers in the mesh are separated in the z axis: layer 1 (z=0  mm to z=−14  mm) represents the skin, scalp, and cerebral spinal fluid (CSF), layer 2 (z=−14  mm to z=−16  mm) represents gray matter (cortex), and layer 3 (z=−16  mm to z=−30  mm) represents white matter (deep brain tissue).

The thickness of each layer was chosen based on the average anatomy from previous related studies. The skin and scalp thickness of 14 mm was derived from the sum of the average scalp and skin thickness (6.9 and 6.0 mm, respectively) from Strangman et al.,^23^ where they segment the Colin27 head model. The thickness of the gray matter layer (cortex) was chosen as 2 mm as derived from Ref. 24, where Fischl and Dale segmented cortex thicknesses from MRI images of 30 subjects and obtained an average gray matter thickness of 2.5 mm, which for this work is rounded down to 2 mm. Finally, the third layer, white matter thickness was chosen to be 14 mm, so overall the mesh had a depth of 30 mm [Fig. 1(b)].

The optical properties, presented in Table 1, were averaged from various studies at 690, 750, 780, and 830 nm^25^^–^^27^ found in Ref. 23, so they represent a realistic range of NIRS wavelengths. For layer 1, a weighted average of the optical properties for the skin, scalp, and CSF were taken based on their average thickness.^23^ Layers 2 and 3 are the optical proprieties of gray matter and white matter, respectively.

**Table 1:** The absorption and reduced scattering coefficients for each layer in the FEM.^23^^,^^25^^–^^27^

	Absorption coefficient (${\mathrm{mm}}^{-1}$)	Reduced scattering coefficient (${\mathrm{mm}}^{-1}$)
Layer 1	0.0139	0.755
Layer 2	0.0195	1.10
Layer 3	0.0169	1.35

On the surface of the model (z=0  mm), 24 sources (blue crosses) and 28 detectors (red circles) are modeled, which are arranged in the conventional “high density” arrangement,^9^^,^^10^ with nearest-neighbor (NN) distances between a given source and multiple detectors of 13.04 mm (NN1), 29.10 mm (NN2), 39.04 mm (NN3), and 46.92 mm (NN4), giving rise to 348 measurement channels. Due to extensive artifacts observed at the edges of the high density grid, a reduced field of view (FOV) is used to present the results of the analysis. This FOV can be seen in Fig. 1(a) as an orange dotted line. The FOV has dimensions of 102 mm in the x axis, 50 mm in the y axis, and is centered at x=y=0  mm.

For analysis, there are three types of simulated data used: CW (amplitude only), FD (phase only), and the combination of the two, CW&FD (amplitude and phase). Then, for each of these data types, there are three modulation frequencies used, defined as 78.125, 140.625, and 203.125 MHz (based on ISS Imagent) as well as a combination of the three. To introduce realistic noise into the simulated data, a noise model was taken from Ref. 10. In this, considered resting data were from a subject at 140.625 MHz at 830 and 690 nm. Analysis of the data provided the percentage variation of the log mean intensity and raw phase noise in degrees by calculating the standard deviation of the two signals, respectively, using the same NN1 to NN4 distances as used in this study. The amplitude and phase noise were then averaged from 830 and 690 nm and added to the simulated CW and FD data, respectively. It is assumed for CW data, the noise is constant and that for FD data the noise scales linearly as a function of modulation frequency.

There are four main steps in the developed methodology: (1) calculating the Jacobian (sensitivity matrix), (2) generating simulated measurements, (3) image reconstruction, and finally (4) calculation of performance metrics from the reconstructed images. The presented work will only consider the accuracy for recovering $\mu_{a}$ from boundary measurement, assuming a known scattering property, which can be considered valid for imaging functional vascular-related changes as in human brain fNIRS studies.

Photon propagation can be described using a forward model based on the diffusion approximation,^22^ which assumes that scattering dominates over absorption; after a given number of photon interactions with matter, the photons undergo a random walk, i.e., they are diffusing through the medium. This occurs for pathlengths of a few millimeters or less in biological tissue.^11^ From the forward model, the inverse problem can be solved for which the Jacobian, J (sometimes called the sensitivity or “A” matrix) is used. The Jacobian is a matrix for a given modulation frequency that defines the influence of the spatially varying optical properties ∂X within the model on the change in the measured data as collected for a given source/detector pair, ∂Y. Then, the Jacobian is given as

∂Y=J∂X. (2)

The Jacobian as defined has separate components (kernels) for each data-type (amplitude and phase), resulting from changes in the absorption coefficient ($\mu_{a}$). A different Jacobian is therefore calculated for each modulation frequency for each data-type (amplitude/phase). The dimension of the Jacobian is defined as the number of measurements pairs by number of nodes within the model. From this sensitivity matrix, simulated data were calculated using Eq. (3).

Activations were made at three discrete depths of −14, −15, and −16  mm, respectively. Specifically, target activations are defined by creating a matrix, $X_{\text{sim}}$, [dimensions of number of nodes in the entire model × number of required activation (at a given node)]. Each element of $X_{\text{sim}}$ is then set to zero apart from a point activation, which is set to 10.5. An activation of magnitude 10.5 is used as it causes a maximum of a 5% change in measured CW data at 141 MHz, which is as observed from *in vivo* experiments of visual cortex activations.^28^ Simulated functional measurements, $Y_{\text{sim}}$, are then generated as

$$Y_{\text{sim}}=JX_{\text{sim}}.$$ (3)

Using the generated data from single target activation, image reconstruction can be performed. In this work, no noise has been added to the simulated data, as the aim is to evaluate the effect of specific data-types in image reconstruction. Previous work has utilized a realistic measure of the noise to simulated data from a multilayered head model and demonstrated that the inclusion of phase measurements does improve image recovery as compared to intensity measurements alone.^10^ Following from Eq. (3), a Moore–Penrose pseudoinverse^29^ with Tikhonov regularization is used to approximate the inverse of the Jacobian, $J^{-1}\approx J^{\#}$. $J^{\#}$ is given as

$$J^{\#}=J^{T}{\left(JJ^{T}+\alpha I\right)}^{-1},$$ (4)

where I is an identity matrix and α is the regularization term, given as

$$\alpha =\lambda *\max[\mathrm{diag}(JJ^{T})],$$ (5)

where λ is the regularization parameter chosen to be 0.01 for both CW and FD simulated data. Using the pseudoinverse Jacobian, the reconstruction of changes in optical properties $X_{\mathrm{recon}}$ is as

$$X_{\mathrm{recon}}=J^{\#}Y_{\text{sim}}.$$ (6)

For each point activation at a single depth in the HD grid FOV, three-dimensional spatially varying image reconstruction is performed (at 5253  nodes×3  depths in z). To calculate the performance metrics for given activation point within the cortex, only nodes within the cortex layer [layer 2 in Fig. 1(b)] were considered for analysis.

**Fig. 2:**
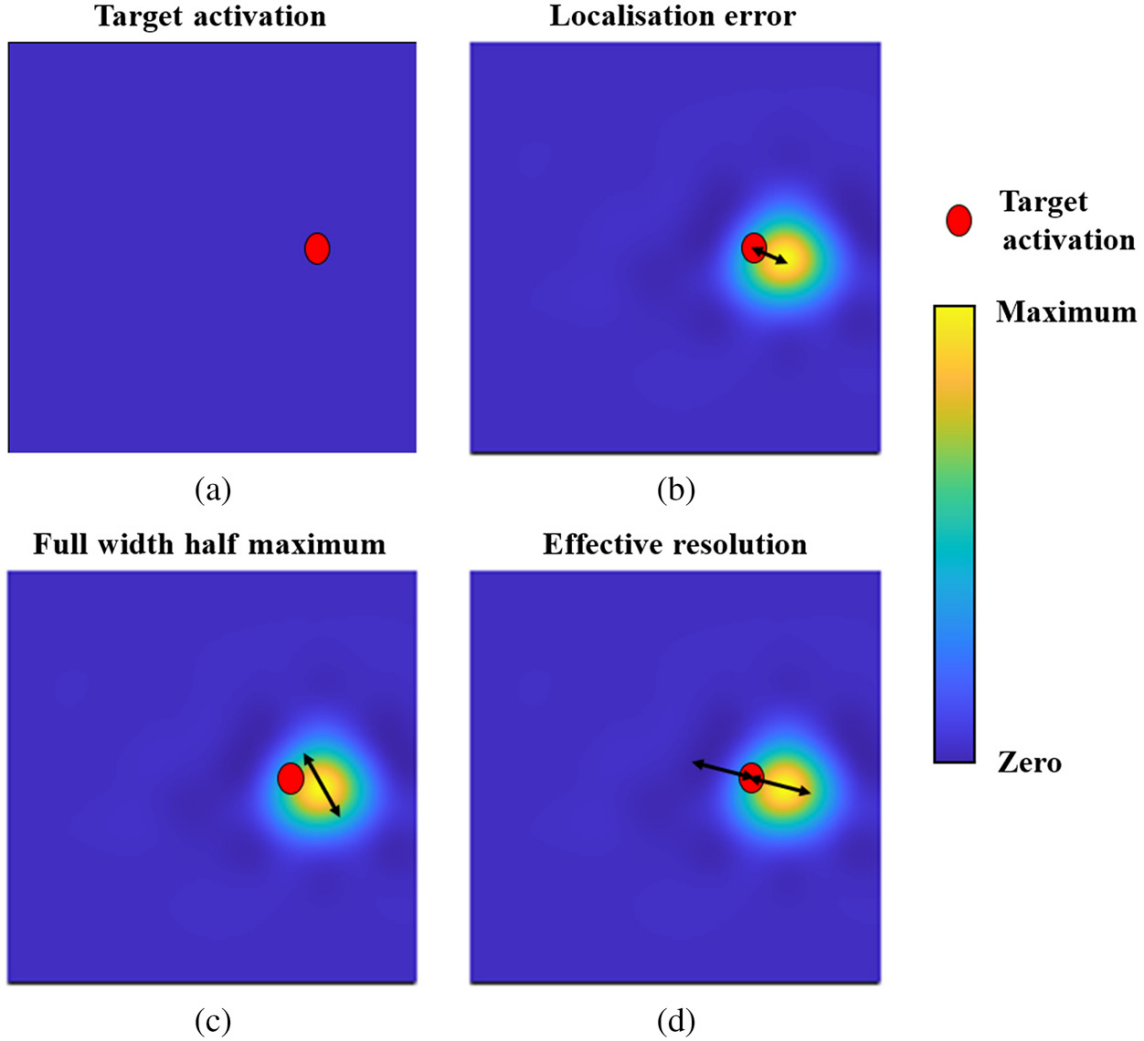
A diagram of how each performance metric is calculated. (a) A single node is set to 10.5 (activated), whereas all other nodes are set to zero. Image reconstruction is then performed everywhere. Only nodes within the cortex are considered for the performance metrics. (b) The LOCA is given by the distance between the target activation and the maximum recovery of the reconstruction. (c) The FWHM is given by the maximum distance between any two nodes that are more than or equal to 50% of the maximum of the reconstruction. (d) The ERES is twice the maximum distance between the target activation and any node that is more than or equal to 50% of the maximum of the reconstruction.

The choice of performance metrics is adapted from the work of White and Culver in 2010 to allow a direct comparison with established standards.^15^ Specifically, these are the localization error (LOCA), the full-width half-maximum (FWHM), and the ERES, which are used to quantify how accurate image reconstructions are, particularly in the context of cortical activation. A description of how the three metrics are calculated is given in Fig. 2.

LOCA is given by the distance between the target activation and the location of the recovered maximum of the reconstruction. This represents the minimum spatial error between where cortical activation occurs and where the tomographic reconstruction places the maximum of the recovery. FWHM is given by the maximum distance between any two nodes in the reconstruction that are more than or equal to 50% of the maximum of the reconstruction. ERES is twice the maximum distance between the target activation and any node in the reconstruction that is more than or equal to 50% of the maximum of the reconstruction. ERES combines the concept of mislocalization, as well as recovering to be bigger than it is. Therefore, it represents a more realistic performance metric.

**Fig. 3:**
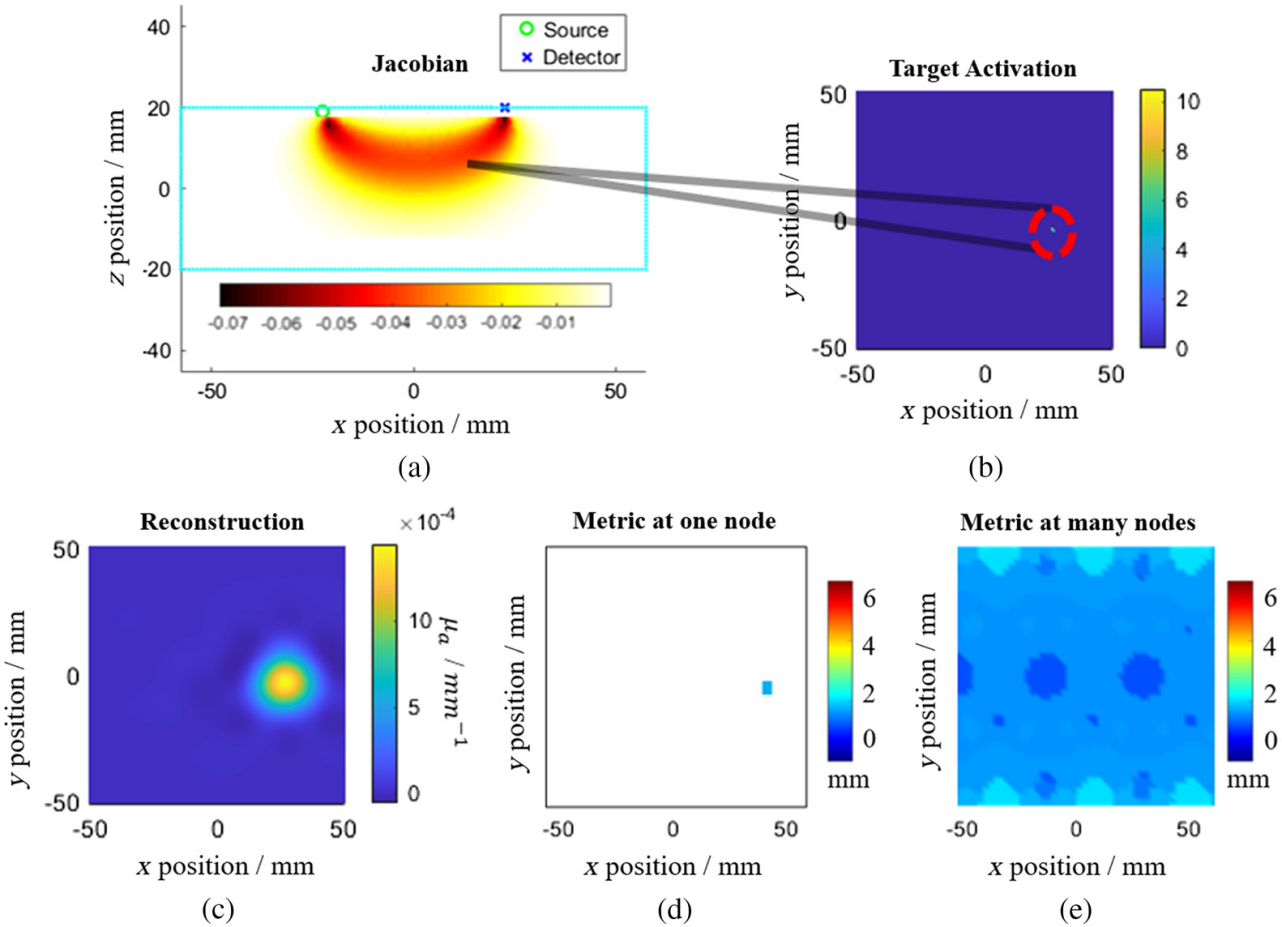
The processes involved in generating the performance metrics. (a) A Jacobian (mm) is shown for a 2D FEM with one source and one detector. This shows the sensitivity of a measurement being made at detector 1 from source 1, due to a point activation at that node. (b) Each node is then activated or perturbed by setting the node to 10.5, which would cause a maximum of a 5% change in CW data at 141 MHz.^28^ (c) From the point activation, image reconstruction is performed at every node within the mesh. (d) Each performance metric is then calculated by considering nodes within the cortex. (e) Steps (b)–(d) are then repeated for every node within the cortex layer. The average value and standard deviation of each performance metric can then be calculated.

To calculate each performance metric, image reconstruction was performed that has been described above and summarized in Figs. 3(a)–3(c).

After image reconstruction, the metrics are calculated for each node for a single depth within the cortex layer of the model. There are 5253 nodes for a given depth and 15,759 nodes for the cortex layer. Then, the average value of a given metric is calculated individually at three individual layers at −14, −15, and −16  mm, respectively, as well for the entire cortex layer (averaged across all depths). The entire model is used for image reconstruction. Then, only nodes within the cortex layer are used for metric calculations.

**Fig. 4:**
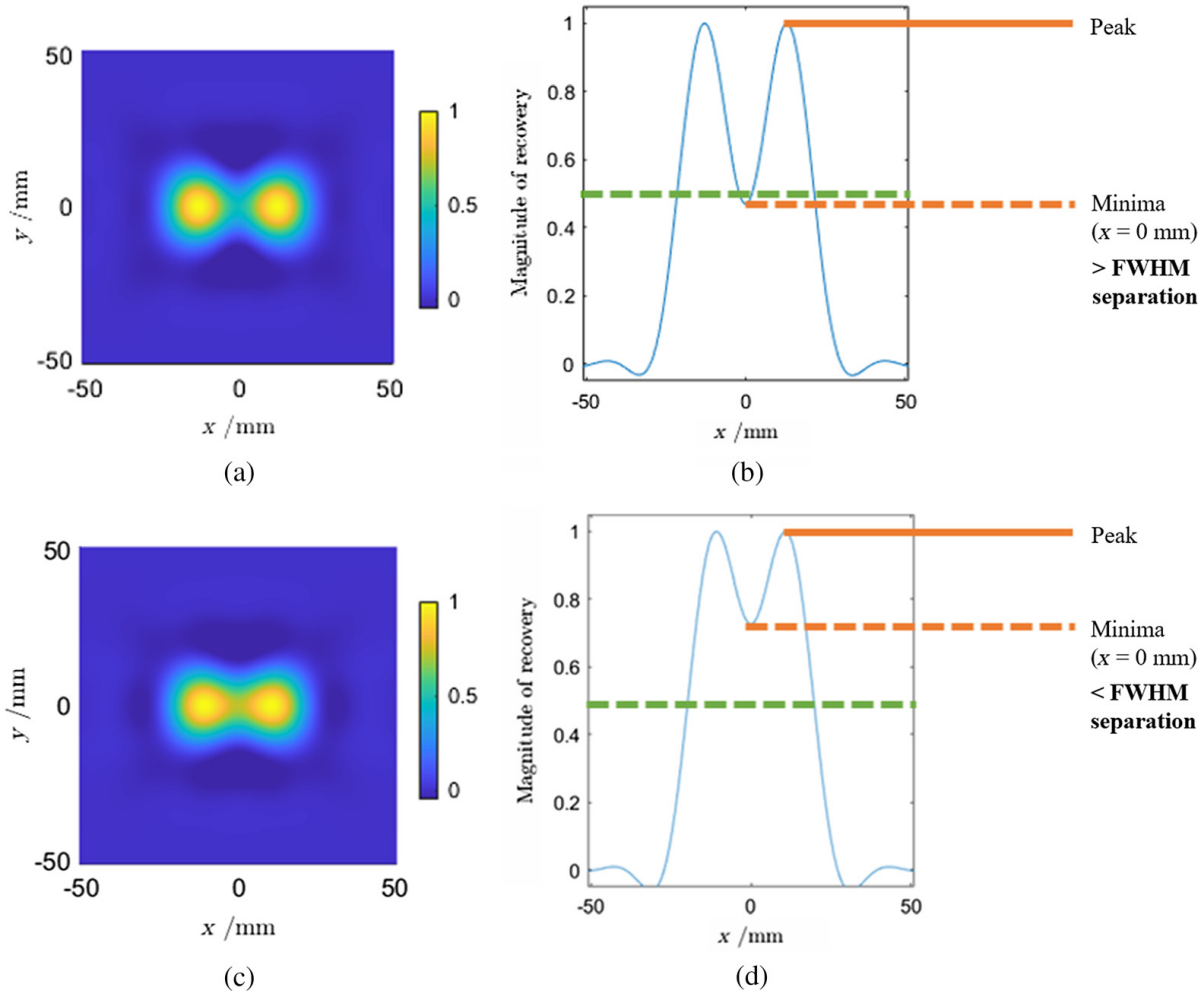
Diagrams showing the separation of two point activations. (a) Image reconstruction of two point activations at ±13  mm, respectively, in the x axis. The reconstructions are summed from the two activations and normalized. (b) A graph showing the magnitude of the reconstruction along the x axis at y=0  mm. The graph shows that there is an FWHM separation since the minima is below 50% of the maximum recovery. (c) As described for (a), but the two activations are at ±11  mm, respectively. (d) As described for (b), but there is now less than FWHM separation. This is because the minima is above 50% of the maximum recovery.

To evaluate the resolving power of the different data types and modulation frequencies, two point perturbations were simulated at a depth of −15  mm (middle of the cortex layer). They were placed along y=0  mm and at ±X  mm in the x axis. Figure 4 shows two examples of point activation as separated by ±13  mm (a and b) and ±11  mm (c and d). The recovered activations are normalized so that the maximum is unity. A metric to determine how close the two point activations can be before being unable to be identified as two separate points is by considering the minimum of the recovery profile to be greater than 0.5 (i.e., at FWHM). Other metrics to determine separation are looking at a 3 dB or 0.707 of the maximum value separation. If the minimum of the recovery profile is <0.707 then the two activations may be considered as separated. Any threshold used to determine separation can be argued as arbitrary and fundamentally is subjective. For example, Figs. 4(c) and 4(d) show a separation that is less than the FWHM separation; however, it could be argued that the two activations can still be separated. A lower value at the minima means that the two points are easier to resolve. For this analysis, FWHM (0.5) and 3 dB (0.707) separation are considered.

## 3 Results

The results of the three performance metrics, LOCA, FWHM, and ERES are shown and discussed in the following sections. In Secs. 3.1 to 3.3, the results shown are averaged from point activations across the cortex layer (Z=−14  mm to Z=−16  mm) within the FOV as shown in Fig. 1(a). Section 3.4 shows results in each layer of the cortex individually within the FOV as shown in Fig. 1. Finally, Sec. 3.5 provides numerical results from the dual point spread function analysis at a depth of Z=−15  mm.

### 3.1 CW versus FD versus CW and FD

**Fig. 5:**
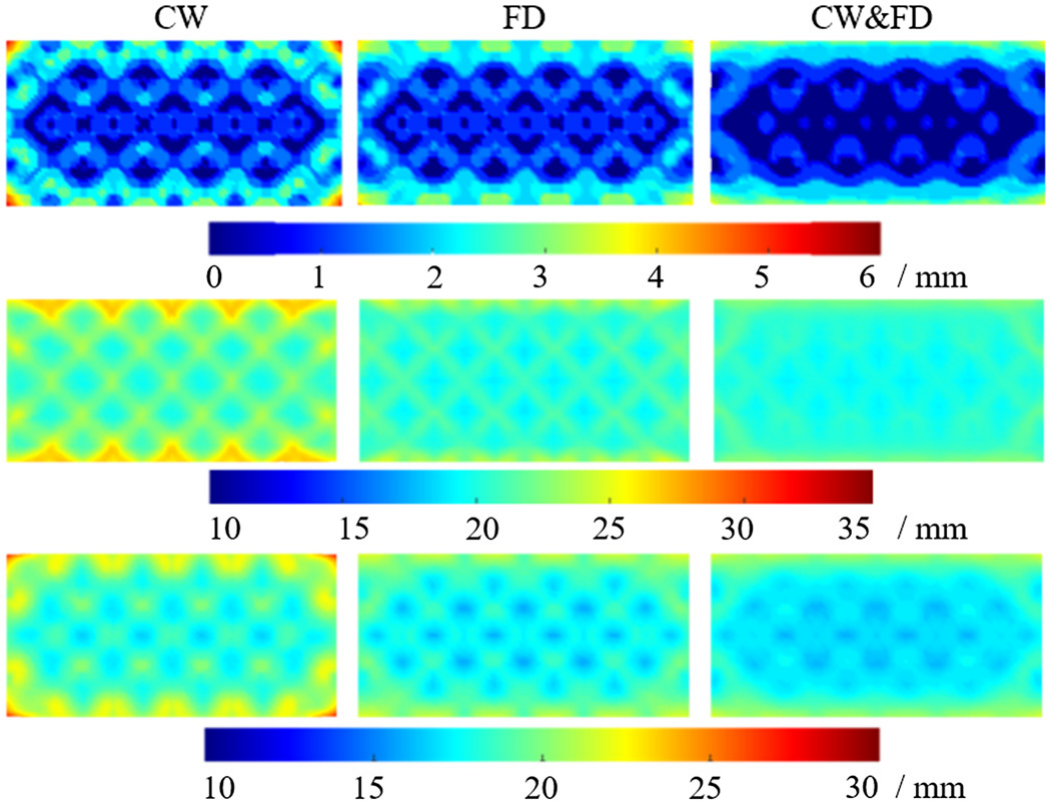
The LOCA (first row), FWHM (second row), and ERES (third row) are shown at 140.625 MHz for the CW, FD, and CW and FD case, respectively. The graphs are the average of point activations within the cortex layer from a depth of −14 to −16  mm and within the FOV shown in Fig. 1(a).

Figure 5 shows the LOCA, FWHM, and ERES for CW, FD, and the combined CW and FD simulated data, respectively. This was performed at a modulation frequency of 140.625 MHz (referred to as 141 MHz). The average value of each metric is shown in Table 2. For the LOCA, FD performs 12.78% better than CW alone, and the combined measurement of CW and FD is 47.05% better than CW alone. The combined CW and FD measurement for LOCA also results in a more uniform central region than either CW or FD, as well as less artifacts around the edge of the FOV. This is particularly seen on the left- and right-hand side of the FOV. It is only in the CW case that the LOCA dramatically increase to a maximum of 5.55 mm in the corners. For comparison, the FD and CW and FD measurements reach a maximum of 3.74 and 4.03 mm, respectively.

**Table 2:** The average and standard deviation of the LOCA, FWHM, and ERES for the CW, FD, and CW and FD measurements. Rows one to three are without noise and rows four to six are with noise. These are at 140.625 MHz and are the average of point activations in the cortex layer from a depth of −14 to −16 mm within the FOV shown in Fig. 1(a). These results can be seen in Fig. 5.

	Metric	CW	FD	CW and FD
Noise free	LOCA/mm	1.50±0.90	1.33±0.83	1.02±0.90
	FWHM/mm	19.96±1.42	18.70±0.83	18.36±0.63
	ERES/mm	21.64±1.97	20.43±1.50	19.78±1.52
Noise added	LOCA/mm	1.50±0.90	1.79±0.94	1.46±0.80
	FWHM/mm	19.97±1.42	20.96±5.80	19.30±2.09
	ERES/mm	21.66±1.97	24.94±11.31	21.67±4.10

For the FWHM, FD performs 6.73% better than CW and the combined measurement of CW and FD is 8.71% better than CW alone. While the magnitude of performance increases by combining CW and FD is less than shown with LOCA, the standard deviation of the FWHM drastically reduces. The FD case has a 71.08% lower standard deviation than CW, and the CW and FD case has a 125.39% lower standard deviation than CW. This can be seen visually, with the FWHM– CW result in Fig. 5 showing artifacts on the top and bottom of the FOV.

The FD and combined measurement of CW and FD have greatly reduced artifacts in these areas. For the ERES, FD performs 5.92% better than CW and the combined measurements are 9.40% better than CW alone. For the ERES, the FD and combined measurements have lower artifacts around the edges of the FOV and a more uniform center. In particular, the FD and combined measurements have standard deviations, which are ∼30% lower than that of the CW measurements.

The inclusion of noise can be seen in rows four to six of Table 2. In the noise added model, FD measurements perform worse as compared to the CW measurements. However, despite the decreased average performance of FD alone, the combined CW and FD performs better than either CW or FD alone for the LOCA and FWHM. For the ERES, CW and FD performs marginally worse than CW alone.

### 3.2 Effect of Modulation Frequency

**Fig. 6:**
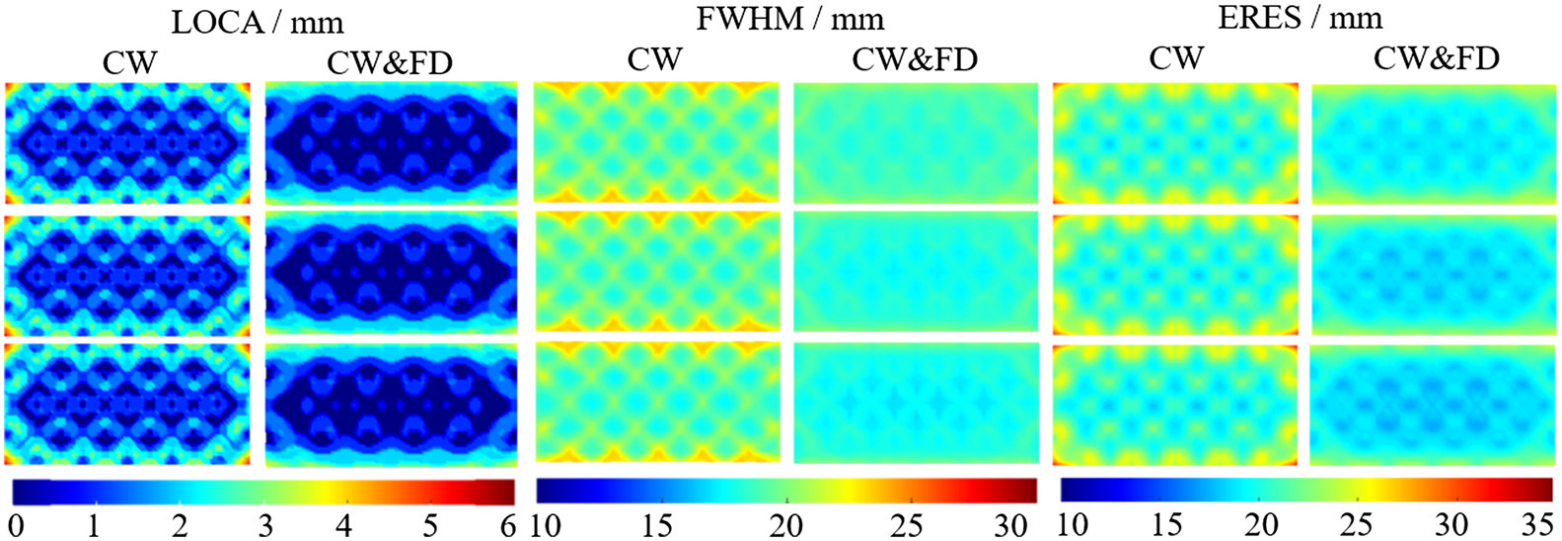
The LOCA, FWHM, and ERES are shown at 78 MHz (first row), 141 MHz (second row), and 203 MHz (third row), respectively. For each modulation frequency, the CW and CW and FD measurements are shown. The graphs are the average of point activations within the cortex layer from a depth of −14 to −16  mm and within the FOV shown in Fig. 1(a).

The LOCA, FWHM, and ERES were evaluated for three different modulation frequencies of 78.125, 140.625, and 203.125 MHz. For convenience, these will be referred to as 78, 141, and 203 MHz, respectively. Figure 6 shows the three metrics for CW and the combined data types (CW and FD), with the FD results omitted as the trend is similar to those presented above. The average values of these results are shown in Table 3.

**Table 3:** The average and standard deviation of the LOCA, FWHM, and ERES for the CW and CW and FD measurements. These are evaluated at 78 MHz (first row), 141 MHz (second row), and 203 MHz (third row). They are the average of point activations in the cortex layer from a depth of −14 to −16  mm within the FOV shown in Fig. 1(a). These results can be seen in Fig. 6.

LOCA/mm		FWHM/mm		ERES/mm	
CW	CW and FD	CW	CW and FD	CW	CW and FD
1.50±0.89	1.08±0.93	20.11±1.39	18.97±0.62	21.80±1.93	20.40±1.58
1.50±0.90	1.02±0.90	19.96±1.42	18.36±0.63	21.64±1.97	19.78±1.52
1.49±0.90	1.02±0.88	19.75±1.42	18.04±0.65	21.41±2.04	19.46±1.50

As expected from the single modulation frequency case in Fig. 5, the combined measurement performs better than the CW measurement across all three modulation frequencies. Increasing the modulation frequency increases the performance of both CW and CW and FD for each metric. In the case of the LOCA, this improvement is smaller, with a 0.67% (0.01 mm) change for CW and a 5.88% change for CW&FD case. For the FWHM, going from 78 to 141 MHz causes an 1.82% and 5.15% increase in performance for CW and CW and FD, respectively. A significant increase in performance of 11.47% is shown when considering the difference between CW at 78 MHz to CW and FD at 203 MHz. For all modulation frequencies, the CW and FD case shows less artifacts around the top and bottom of the FOV than CW. The standard deviation of FWHM stays near constant across modulation frequencies, with the CW and FD measurements showing a 125.39% decrease in standard deviation compared to CW. For the ERES, there is a 1.82% increase for CW measurements from 78 to 203 MHz and a 4.83% increase for CW and FD measurements. Again, the most significant increase in performance occurs when considering both increasing modulation frequency and use of CW and FD combined measurements instead of just CW measurements of 12.02%.

### 3.3 Combining Modulation Frequencies

**Fig. 7:**
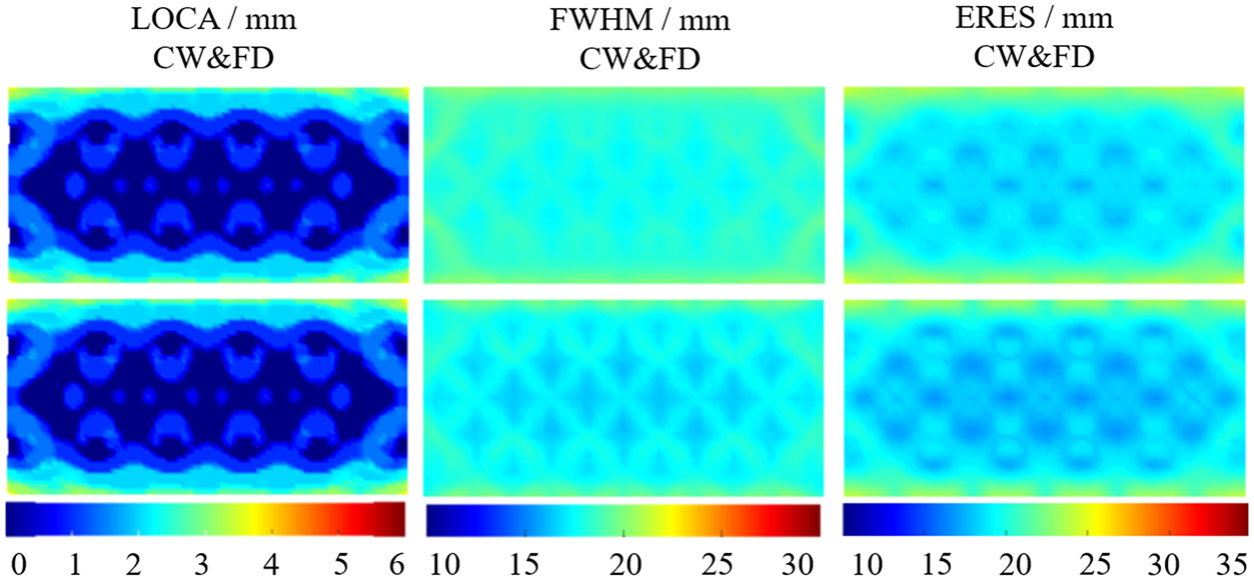
The LOCA, FWHM, and ERES are shown using 140.625 MHz (first row) and the combined 78.125, 140.625, and 203.125 MHz (second row) modulation frequencies. The CW and FD measurements are shown. The graphs are the average of point activations within the cortex layer from a depth of −14 to −16  mm and within the FOV shown in Fig. 1(a).

The LOCA, FWHM, and ERES were evaluated using 140.625 MHz and then by combining measurements at all three frequencies. Figure 7 shows the three metrics evaluated using CW and FD measurements. CW measurements were omitted as the CW contribution to three combined modulation frequency measurements were calculated to be the same as reference modulation frequency of 141 MHz. The average of these results is shown in Table 4.

**Table 4:** The average and standard deviation of the LOCA, FWHM, and ERES for CW and FD measurements. These are evaluated at a single frequency, 140, 625 MHz, and three combined frequencies 78.125, 140.625, and 203.125 MHz. Rows one and two are without noise and rows three and four are with noise. They are the average of point activations in the cortex layer from a depth of −14 to −16  mm within the FOV shown in Fig. 1(a). These results can be seen in Fig. 7.

	Modulation frequency/MHz	LOCA/mm (CW and FD)	FWHM/mm (CW and FD)	ERES/mm (CW and FD)
Noise free	141	1.02±0.90	18.36±0.63	19.78±1.52
	78 and 141 and 203	1.09±0.87	17.64±0.72	19.09±1.54
Noise added	141	1.46±0.80	19.30±2.09	21.67±4.10
	78 and 141 and 203	1.39±0.79	18.27±1.37	20.38±2.68

For the LOCA, there is a 6.86% performance decrease combining three modulation frequencies as compared to the single frequency case. The FWHM and ERES show an increase in performance due to combining the modulation frequencies. For the FWHM, using three modulation frequencies is 4.68% (0.72 mm) better than the single frequency case. The minimum FWHM value of the three combined modulation frequencies is 16.56 mm compared to 17.31 mm for the ERES, the performance increase is 3.61% (0.69 mm). Although the FWHM decreases with combining modulation frequencies, the standard deviation increases by 14.28% (0.09 mm) and remains near constant for the ERES. Overall the lowest FWHM and ERES are found using the three combined modulation frequencies using the combined CW and FD data.

The inclusion of noise can be seen in rows three and four of Table 4. In the noise added model, the combination of the three modulation frequencies increases imaging performance in each of the three metrics. This is 4.8% (0.07 mm) for the LOCA, 5.4% (1.03 mm) for the FWHM, and 6.0% (1.29 mm) for the ERES. The combination of three modulation frequencies also decreases the standard deviation across the three metrics. This is most noticeable for the ERES, where the decrease is 34.6% of the three modulation frequencies compared to the single case.

### 3.4 Performance Across Depth of Cortex

**Fig. 8:**
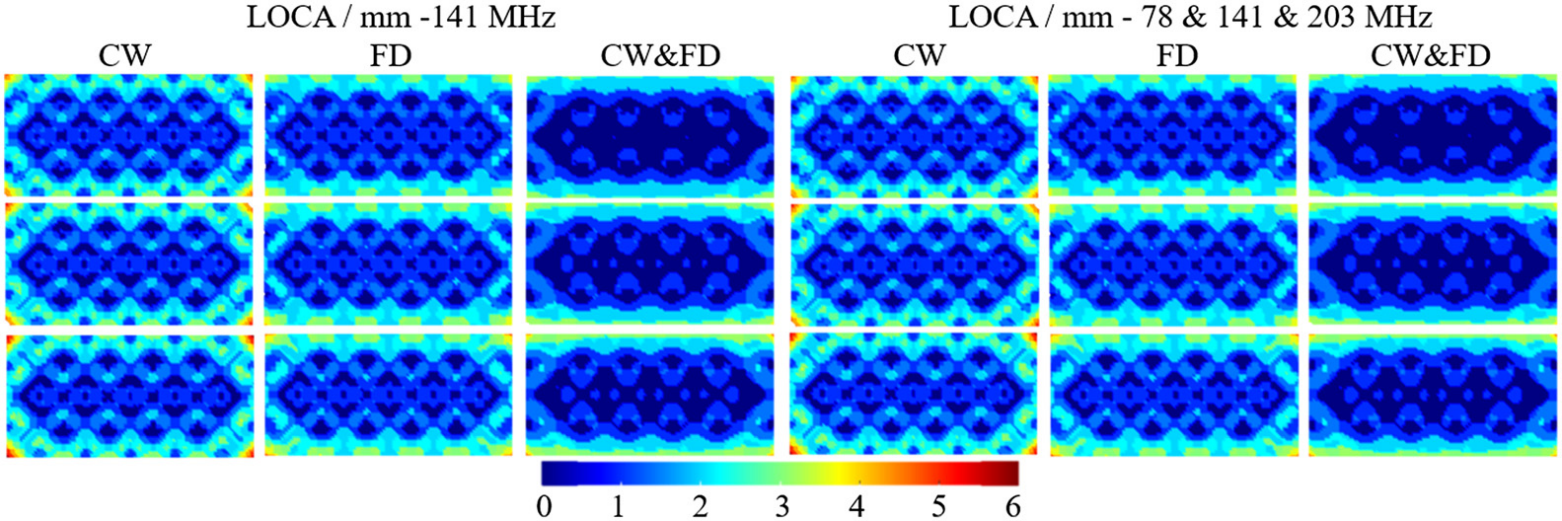
The LOCA for CW, FD, and CW and FD at 141 MHz (left three columns) and the combined 78 and 141 and 203 MHz (right three columns). The first row is at a depth of 14 mm, the second row is at a depth of 15 mm, and the bottom row is at a depth of 16 mm.

In Secs. 3.1–3.3, the results of the LOCA, FWHM, and ERES were shown as the average across the depth of the cortex (−14 to −16  mm); however, in this section, the metrics are evaluated at each discrete depth in the cortex, namely at −14, −15, and −16  mm. CW, FD, and CW and FD data types are evaluated at 141 MHz and the combined 78 and 141 and 203 MHz. The LOCA gets worse as a function of depth and is seen in all data types and modulation frequencies as shown in Fig. 8 and Table 5. As expected, the combination of CW and FD data performs better than just FD and CW alone. This is across both the single 141 MHz and combined 78 and 141 and 203 MHz modulation frequencies, respectively. For 141 MHz, the LOCA at −14  mm for CW is worse than that of FD at −16  mm. Similarly, the LOCA at −14  mm for FD is worse than that of CW and FD at −16  mm. This shows that FD has a better LOCA at higher depths than CW and the combined data type does even better than FD at higher depths within the cortex. The combined data type performs worse in the combined modulation frequency case than for the single modulation frequency. However, the decrease in performance is 6% of the standard deviation of both of these values. This may not be considered significant.

**Table 5:** The average and standard deviation of the LOCA for CW, FD, and CW and FD at 141 MHz (left three columns) and the combined 78 and 141 and 203 MHz (right three columns). They are the average of point activations within a single layer of the FEM. These results can be seen in Fig. 8.

Activation depth/mm	LOCA/mm (141 MHz)			LOCA/mm (78 and 141 and 203 MHz)		
	CW	FD	CW and FD	CW	FD	CW and FD
−14	1.46±0.88	1.21±0.83	0.87±0.90	$1.46±0.88	1.10±0.87	0.93±0.87
−15	1.50±0.91	1.35±0.85	1.04±0.94	$1.50±0.91	1.28±0.92	1.11±0.90
−16	1.53±0.94	1.44±0.89	1.16±0.96	$1.53±0.94	1.38±0.94	1.22±0.92

**Fig. 9:**
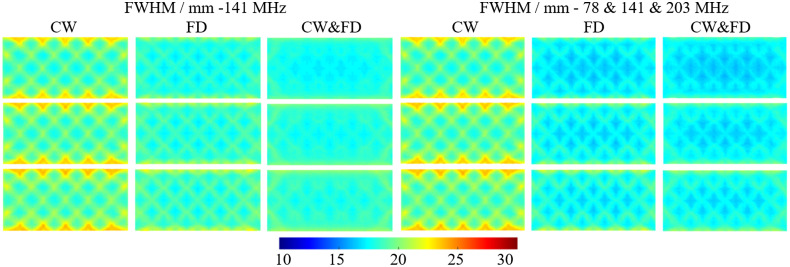
The FWHM for CW, FD, and CW and FD at 141 MHz (left three columns) and the combined 78 and 141 and 203 MHz (right three columns). The first row is at a depth of 14 mm, the second row is at a depth of 15 mm, and the bottom row is at a depth of 16 mm.

The FWHM increases as a function of depth for all data types and modulation frequencies. This is shown in Fig. 9 and Table 6. Again, the CW and FD data type performs better than FD or CW alone in all cases. For 141 MHz, the FWHM at −16  mm of FD is less than that of CW at −14  mm. Similarly, the FWHM of CW and FD at −16  mm is better than FD at −14  mm. This is what is observed for the LOCA; however, for the combined modulation frequency while the FD follows this pattern compared to CW, CW and FD at −16  mm is not better than FD at −14  mm. CW data show the largest standard deviation of FWHM for both the single and multiple modulation frequency by a factor of two compared to FD and CW and FD. This increase in standard deviation can be seen from artifacts on the top and bottom of the FOV in Fig. 9 at each depth in the cortex. These artifacts seen in the FD and CW and FD case, however, are less prominent. Overall, the best FWHM performance is found using the combined modulation frequencies and combined CW&FD data, due to having the lowest average FWHM at each depth, as well as the lowest standard deviation.

**Table 6:** The average and standard deviation of the FWHM for CW, FD, and CW and FD at 141 MHz (left three columns) and the combined 78 and 141 and 203 MHz (right three columns). They are the average of point activations within a single layer of the FEM. These results can be seen in Fig. 9.

Activation depth/mm	FWHM/mm (141 MHz)			FWHM/mm (78 and 141 and 203 MHz)		
	CW	FD	CW&FD	CW	FD	CW&FD
−14	19.73±1.40	18.36±0.74	18.00±0.59	$19.73±1.40	17.47±0.74	17.27±0.63
−15	19.98±1.43	18.72±0.84	18.39±0.64	$19.98±1.43	17.87±0.88	17.65±0.73
−16	20.18±1.44	19.03±0.92	18.39±0.68	$20.18±1.44	18.20±0.99	18.00±0.84

**Fig. 10:**
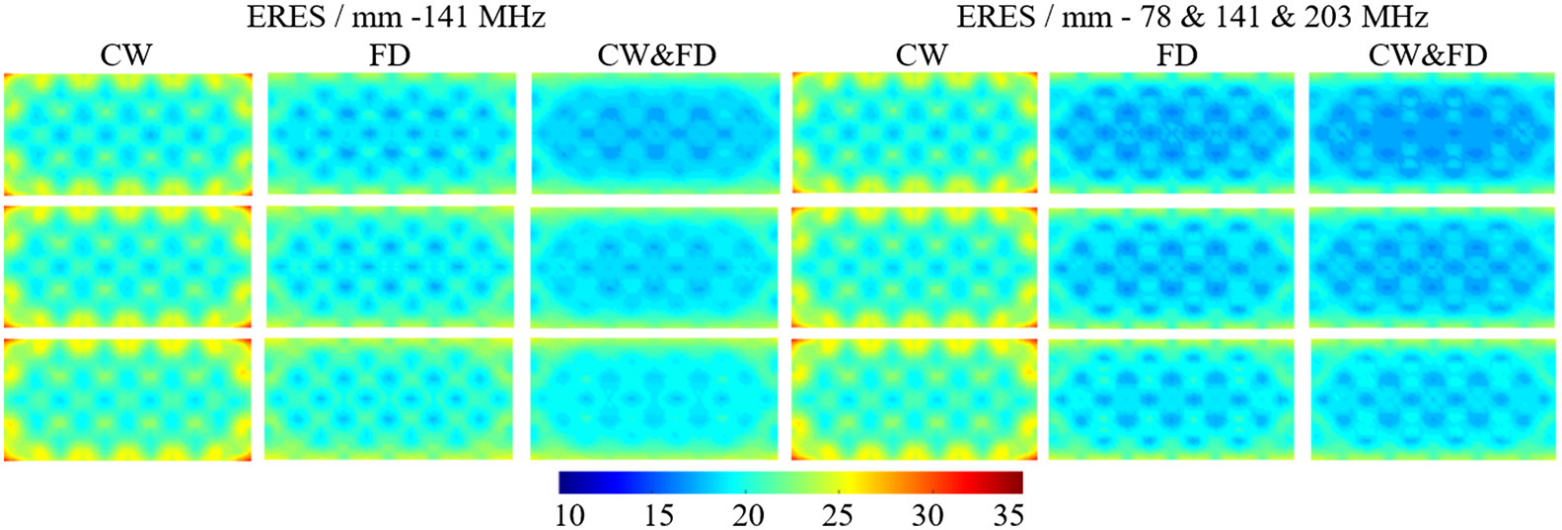
The ERES for CW, FD, and CW and FD at 141 MHz (left three columns) and the combined 78 and 141 and 203 MHz (right three columns). The first row is at a depth of 14 mm, the second row is at a depth of 15 mm, and the bottom row is at a depth of 16 mm.

Similar to the LOCA and FWHM, ERES gets worse as a function of depth. This can be seen in Fig. 10 and Table 7. For 141 MHz, FD measurements offer a modest increase in performance compared to CW (6% to 7%). CW and FD performs slightly better (3%) than FD throughout the cortex. Using three modulation frequencies offer further performance increase. The ERES at −14  mm using three modulation frequencies is 0.72 mm or 3.88% better than using 141 MHz alone. At −16  mm, the benefit remains similar. Multimodulation frequency is 0.64 mm or 3.25% better than 141 MHz alone. The artifacts seen in the FWHM (Fig. 9) are also seen for the ERES (Fig. 10), however, are less prominent on the top and bottom of the FOV. There are artifacts seen on the left and right edges of the FOV not seen with the FWHM. These artifacts are seen the most in the CW case, which is reflected in the standard deviation of the FWHM and is ∼25% lower for FD and CW and FD (1.5 mm) compared to CW (2.0 mm).

**Table 7:** The average and standard deviation of the ERES for CW, FD, and CW and FD at 141 MHz (left three columns) and the combined 78 and 141 and 203 MHz (right three columns). They are the average of point activations within a single layer of the FEM. These results can be seen in Fig. 10.

Activation depth/mm	ERES/mm (141 MHz)			ERES/mm (78 and 141 and 203 MHz)		
	CW	FD	CW and FD	CW	FD	CW and FD
−14	21.23±1.94	19.90±1.47	19.26±1.53	21.23±1.94	18.91±1.58	18.54±1.54
−15	21.61±1.99	20.38±1.52	19.75±1.53	21.61±1.99	19.40±1.61	19.04±1.55
−16	22.10±2.00	20.99±1.55	20.33±1.52	22.10±2.00	20.03±1.63	19.69±1.55

### 3.5 Dual Activation Point Spread Function Analysis

For this analysis, CW, FD, and CW and FD data types were used at 141, 203 MHz, and the combined 78, 141, and 203 MHz. For all data types and modulation frequencies, decreasing the separation increases the minimum value of the center of the recovery. This can be seen in Fig. 4, which shows that a smaller separation [Fig. 4(c)] leads to a higher minima [Fig. 4(d)]. Decreasing the separation to zero causes the minimum value to tend to 1 (i.e., not separated at all). At 141 MHz, FD has lower minimum values of the central minima than CW by about 25% at a separation of ±15  mm. The combined CW and FD is 36% lower than CW. Increasing the modulation frequency to 203 MHz increases the resolving power. For FD, 3 dB separation is lost after a separation of ±12  mm at 141 MHz, whereas at 203-MHz 3-dB separation is lost at ±11  mm. This is a net 2 mm difference. The difference between FD and FD and CW is smaller than the difference between changing modulation frequencies. Therefore, increasing modulation frequency or even combining them yields the greatest increase in resolving power. For example, using CW and FD data at a separation of ±12  mm yields a separation of 0.59 at 141 MHz, 0.55 at 203 MHz, and 0.49 at the three combined modulation frequencies. At ±11  mm with CW and FD data, 3 dB separation is not achieved at 141 MHz (0.72) but is with 203 MHz (0.69) and the combined frequencies (0.62).

At ±10  mm, the minimum separation at 141 and 203 MHz converges for CW, FD, and CW and FD. There is only a 4.5% difference between CW (0.89) and CW and FD (0.85). However, at the three combined modulation frequencies, there is still a 14.6% difference between CW (0.89) and CW and FD (0.76). At ±9  mm, this difference is a 6.25%. Only at a separation of ±8  mm do the minimum values converge to 0.99. The full results can be seen in Table 8 (141 MHz), Table 9 (203 MHz), and Table 10 (78 and 141 and 203 MHz).

**Table 8:** The minima values between the two PSF separations at 141 MHz. The two PSFs are at y=0  mm, z=−15  mm, and the x co-ordinates are given in row one. The recoveries are normalized so that the maximum is unity across a given modulation frequency.

x co-ordinates/± mm	15	14	13	12	11	10
CW	0.4116	0.5010	0.5967	0.6966	0.7968	0.8907
FD	0.3013	0.3945	0.4986	0.6116	0.7303	0.8463
CW and FD	0.2649	0.3591	0.4681	0.5914	0.7255	0.8593

**Table 9:** As Table 8 but at 203 MHz.

x co-ordinates/± mm	15	14	13	12	11	10
CW	0.3864	0.4760	0.5729	0.6752	0.7791	0.8778
FD	0.2620	0.3538	0.4581	0.5739	0.6992	0.8265
CW and FD	0.2408	0.3315	0.4373	0.5583	0.6922	0.8308

**Table 10:** As Table 8 but at the combined 78 and 141 and 203 MHz.

x co-ordinates/± mm	15	14	13	12	11	10
CW	0.4116	0.5010	0.5967	0.6966	0.7968	0.8907
FD	0.1836	0.2727	0.3758	0.4922	0.6212	0.7582
CW and FD	0.1845	0.2716	0.3737	0.4916	0.6244	0.7667

**Fig. 11:**
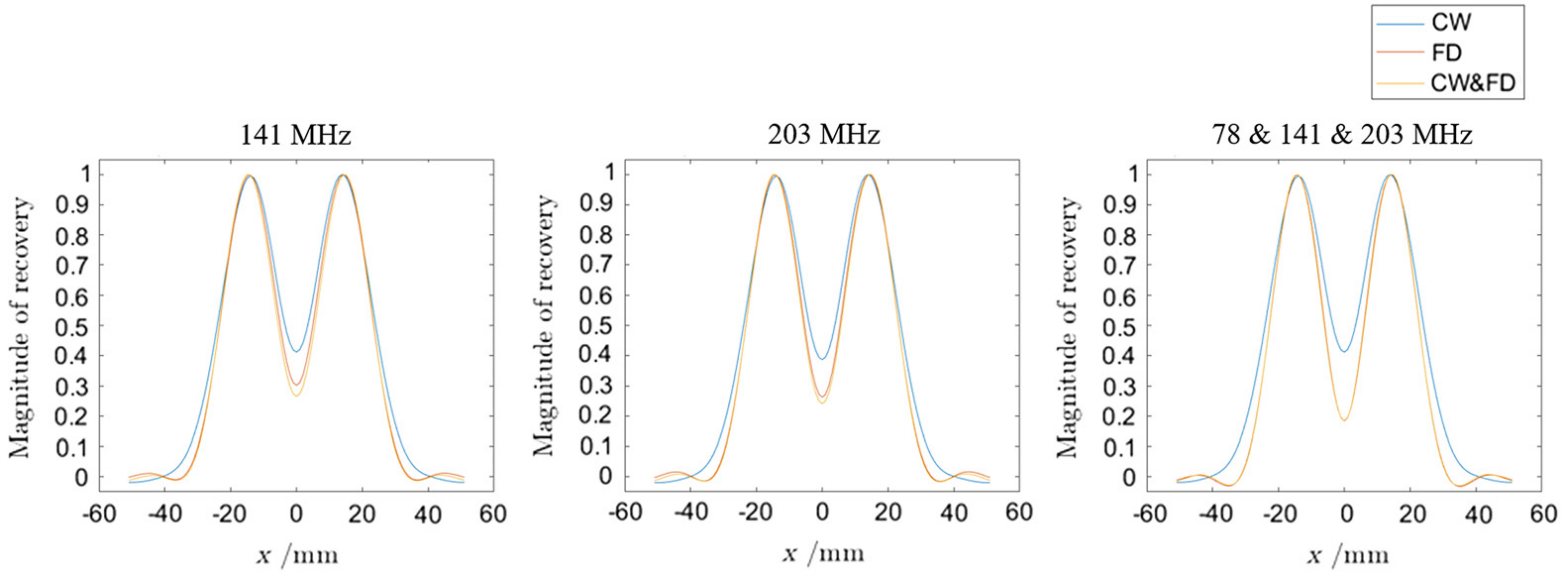
The minima values between the two PSF separations at 141 MHz, 203 MHz, and the combined 78 and 141 and 203 MHz. The CW part of the data is at 141 MHz only. The two PSFs are at x=±15  mm, y=0  mm, and z=−15  mm. The recoveries are normalized so that the maximum is unity across a given modulation frequency.

For visual comparison, the minima values between the two PSFs at a separation of ±15  mm at 141 MHz, 203 MHz, and the combined 78 and 141 and 203 MHz can be seen in Fig. 11.

## 4 Discussions

This work shows that the combination of CW and FD data performs better than CW alone. This agrees with previous work done by Doulgerakis et al.,^10^ which demonstrated that the inclusion of FD data performs better than CW alone in HD-DOT. The data in Fig. 5 and Table 2 show that for each performance metric, FD is between 5% and 12% better than CW alone. Building on previous work, direct evaluation of an HD-DOT style grid is shown for the first time (Fig. 5) between CW, FD, and CW and FD. Not only does CW and FD perform better than FD or CW alone in all metrics, but CW and FD provide more uniform recovery. This is demonstrated with lower standard deviations and visual artifacts around the edges of the FOV. As well as this, CW and FD perform better as a function of depth in the cortex than CW or FD alone and has a better resolving power. This confirms that FD systems should utilize both CW and FD data for the best imaging performance.

The second focus of this study was looking at the effects of changing the modulation frequency of CW and FD measurements, which to date has not been evaluated for HD-DOT. The higher the modulation frequency, the better the imaging performance. This is demonstrated for all metrics for both CW and CW&FD data. The benefit of using higher modulation frequencies is smaller than combining CW and FD data. The biggest gain in performance is when using higher modulation frequencies and CW and FD data. This could be because a higher modulation frequency samples deeper into the model. Sensitivity matrices at 78, 141, and 203 MHz show that there is increased sensitivity further away from the model surface at higher modulation frequencies, which is important for functional cerebral imaging, due to the thickness and effect of superficial tissue.

Another benefit of increased modulation frequency is that the change in measured phased for a given activation increases. For an activation at a depth of 15 mm, there is a 66% increase in the change of measured phase for NN1, between 78 and 203 MHz. The same increase also occurs for second to fourth nearest-neighbor measurements (NN2-NN4). This is due to the deeper sensitivity of phase at higher modulation frequencies.

The biggest impact of this work is the effect of combining multiple modulation frequencies. For nearly all metrics, the best results came from combining FD measurements at different modulation frequencies (78, 141, and 203 MHz) with a CW measurement at 141 MHz. It is expected that this is due to combining varying sensitivity profiles that different modulation frequencies have, which is the same reason why CW and FD performs better than CW or FD alone by providing additional sampling of tissue for a given source and detector pair. Due to the deeper sensitivity of phase data, combining measurements at different modulation frequencies essentially increases sensitivity away from the surface. Based on this work, *in vivo* studies could take multiple phase measurements at different modulation frequencies and couple them with a CW measurement at a single (or multi) modulation frequency to perform HD-DOT with the highest accuracy.

For noise added data at 141 MHz, CW and FD outperforms FD and CW alone, Table 2. However, the inclusion of noise causes the performance of FD data to decrease below that of CW alone. Combining the two datatypes is a compromise between the two, where the better signal-to-noise ratio of CW data is complemented by the increased inherent contrast of FD data as shown in the noise-free model. For the noise added data of the combined 78, 141, and 203 MHz, respectively, the inclusion of three modulation frequencies increases the performance across the three metrics in Table 4. The increased performance is due to the aforementioned reasons in noise-free model. However, what is particularly important in the noise added data is the increased signal-to-noise ratio that three modulation frequencies can provide.

This study reports higher values for FWHM and ERES as compared to previous work such as Ref. 15. For the HD grid, their CW data yield an average FWHM of 12.10 mm and an ERES of 13.50 mm. At 141 MHz, this work has an average FWHM of 19.96 mm and an ERES of 21.64 mm. The reason why the FWHM and ERES are higher with this study is that metrics are formed at a deeper depth of −14 to −16  mm, whereas, in Ref. 15, their analysis is constrained to depths of −5 to −15  mm. Imaging performance becomes worse as a function of depth, as shown in Ref. 10. When the differences in depth are taken into account, the FWHM and ERES in this study become comparable to that in Ref. 15.

For imaging the cortex, the ERES for multimodulation frequency using CW and FD was 18.54 to 19.69 mm (−14 to −16  mm depth). This is still not as good as the resolution of fMRI, which is in the order of a few millimeters^30^ to submillimeter.^31^ However, this work shows that the use of CW and FD measurements, along with combining multiple modulation frequencies can assist current fNIRS methods to gain maximum performance from the modality.

## 5 Conclusions

This work is consistent with previous studies showing that CW and FD data offer significantly better imaging performance than CW alone as applied in fNIRS and HD-DOT. It has shown that CW and FD is better than FD alone, which in turn is better than CW alone. Using higher modulation frequencies for FD measurements cause an increase in performance. This also causes a higher SNR for phase measurements at the expense of lower SNR for CW measurements. However, since phase data have a deeper sensitivity, this is preferable. Combining measurements at multiple modulation frequencies yields increased imaging performance. The next step would be to use CW and FD measurements with multiple modulation frequencies *in vivo* to see if the theoretical gain in performance translates to real life practice.

## Code, Data, and Materials Availability

The Matlab script used to generate and analyze the data in this paper can be made available on request to the first author. The open source NIRFAST toolbox was used to generate the mesh’s, Jacobians, and perform image reconstruction.
